# 1815. Assessment of Therapy Duration for Non-Staphylococcal Gram-Positive Bloodstream Infections

**DOI:** 10.1093/ofid/ofac492.1445

**Published:** 2022-12-15

**Authors:** Alyssa P Gould, Robert L Crawford

**Affiliations:** Novant Health, Charlotte, North Carolina; Novant Health Forsyth Medical Center, Winston-Salem, North Carolina

## Abstract

**Background:**

The optimal duration of antimicrobial therapy for non-staphylococcal Gram-positive (GP) bloodstream infections (BSIs) remains unknown. The purpose of this study was to evaluate clinical outcomes in patients with short (6-10 days) vs. long (11-21 days) duration of therapy (DOT) for non-staphylococcal GP BSIs.

**Methods:**

Adult patients with BSI due to *Streptococcus* or *Enterococcus* spp. admitted to a community health system from January 2016 to December 2021 were included in this retrospective chart review. Patients were excluded for polymicrobial BSI, positive culture deemed contamination, infectious source requiring prolonged treatment, DOT < 6 or > 21 days, or transfer, hospice, or death prior to therapy completion. The primary endpoint was 90-day all-cause mortality. Descriptive statistics, chi-square or Fisher’s exact test, and the student t-test were used for data analysis.

**Results:**

Of 3,544 patients identified, 384 were screened and 86 included. There were 71 patients in the long DOT group and 15 patients in the short DOT group. All included patients were on active therapy for the entire DOT. Baseline characteristics were similar except patients in the long DOT group were more likely to have an infectious diseases consultation and a non-pulmonary source of infection (Table 1). All-cause mortality at 90 days was similar between short vs. long DOT groups (0% vs. 3%, p=0.51) (Table 2). The secondary endpoints of 30-day all-cause mortality, 30-day and 90-day BSI recurrence due to the same organism, 30-day readmission, and hospital length of stay were not statistically different between groups (Table 2). The most common pathogen identified in the short DOT group was *Streptococcus pneumoniae* (n=8), and the most common identified pathogens identified in the long DOT group was *Streptococcus agalactiae* (n=22), *Enterococcus faecalis* (n=14), and *S. pneumoniae* (n=13) (Table 3).

**Figure:**
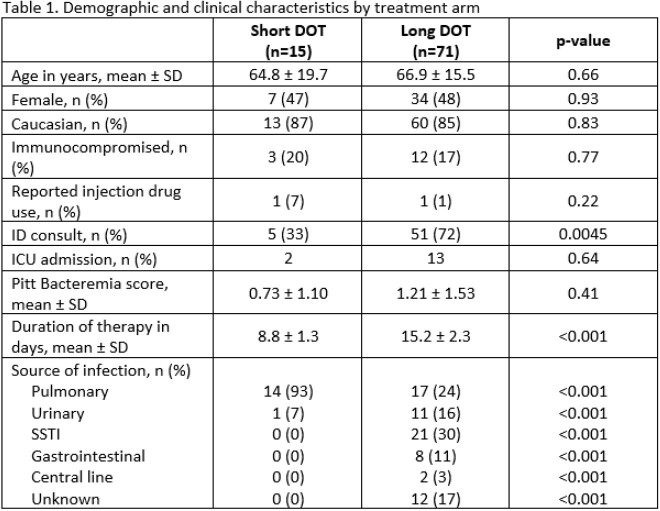

**Figure:**
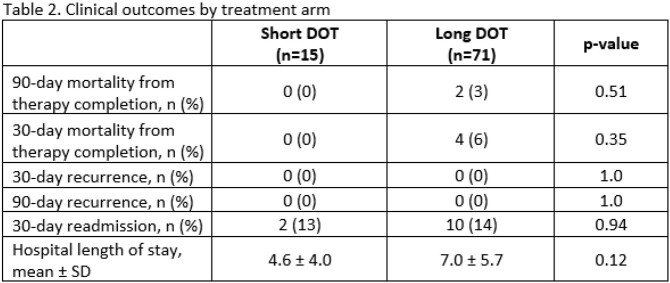

**Figure:**
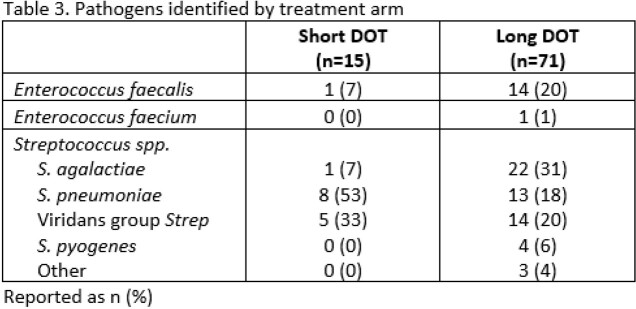

**Conclusion:**

In this retrospective review of non-staphylococcal GP BSIs, there was no statistical difference in clinical outcomes between the short and long DOT arms. Additional research is needed to identify the optimal duration of antimicrobial therapy for non-staphylococcal GP BSIs.

**Disclosures:**

**All Authors**: No reported disclosures.

